# Soymilk Improves Muscle Weakness in Young Ovariectomized Female Mice

**DOI:** 10.3390/nu9080834

**Published:** 2017-08-04

**Authors:** Yuriko Kitajima, Shizuka Ogawa, Shintaro Egusa, Yusuke Ono

**Affiliations:** 1 Musculoskeletal Molecular Biology Research Group, Basic and Translational Research Center for Hard Tissue Disease, Nagasaki University Graduate School of Biomedical Sciences, Nagasaki 852-8588, Japan; mysha.kita@gmail.com; 2 Research and Development Division, Marusanai Co., Ltd., Aichi 444-2193, Japan; shizuka.ogawa@marusanai.co.jp (S.O.); shintaro.egusa@marusanai.co.jp (S.E.)

**Keywords:** soymilk, estrogen, muscle weakness, satellite cells

## Abstract

Estrogens play a key role in an extensive range of physiological functions in various types of tissues throughout the body in females. We previously showed that estrogen insufficiency caused muscle weakness that could be rescued by estrogen administration in a young female ovariectomized (OVX) mouse model. However, long-term estrogen replacement therapy increases risks of breast cancer and cardiovascular diseases. Soymilk contains plant-based protein and isoflavones that exert estrogen-like activity. Here we examined the effects of prolonged soymilk intake on muscle and its resident stem cells, called satellite cells, in the estrogen-insufficient model. Six-week-old C57BL/6 OVX female mice were fed with a dried soymilk-containing diet. We found that prolonged soymilk intake upregulated grip strength in OVX mice. Correspondingly, cross-sectional area of tibialis anterior muscle was significantly increased in OVX mice fed with soymilk. Furthermore, soymilk diet mitigated dysfunction of satellite cells isolated from OVX mice. Thus, these results indicated that prolonged soymilk intake is beneficial for improving muscle weakness in an estrogen-insufficient state in females.

## 1. Introduction

Skeletal muscle is a highly plastic tissue that adapts muscle mass and strength in response to exercise stimuli such as resistance training. In contrast, muscle mass and strength are decreased with denervation, disuse and aging [1,2]. Skeletal muscle characteristics are also influenced by steroid hormones including glucocorticoids, androgens and estrogens [3,4].

In women, estrogens have crucial roles in an extensive range of physiological functions in various types of tissues throughout the body including bone, skeletal muscle, and white-adipose tissues [5]. Studies showed that estrogens maintain muscle strength and promote activation of muscle stem cells, called satellite cells [6,7]. Our previous study also reported that reduced level of estrogens resulted in a decrease in myofiber-sizes and grip strength in ovariectomized (OVX) young female mice [8]. Furthermore, proliferation and differentiation abilities in satellite cells were impaired and indeed muscle regeneration was significantly compromised in OVX mice [8].

Hormone replacement therapy (HRT) is commonly used in postmenopausal women to relive symptoms of menopause. Although muscle mass and strength decline in postmenopausal women, beneficial effects of HRT were shown to preserve muscle contractile function [7,9,10]. The muscle atrophy-related genes such as MAFbx, MuRF-1 and myostatin were downregulated in muscle from postmenopausal women using HRT, which may contribute to maintain muscle function [11]. These data indicated that reducing estrogen levels causes muscle weakness, and HRT can be efficient for maintaining muscle and satellite cell functions in postmenopausal women. However, findings from the Women’s Health Initiative (WHI) Postmenopausal Hormone Therapy Trials and other studies indicate that an extended use of HRT for postmenopausal women leads to an increase in the risks of breast cancer and deep-vein thrombosis [12,13].

Soy products including soymilk exert preferable effects on reducing the risks of cardiovascular diseases and osteoporosis, cholesterol levels, and UV radiation-induced skin inflammation [14,15,16,17,18,19]. Soymilk contains enriched plant proteins, essential amino acids, polyunsaturated fatty acids, vitamins and minerals. Soymilk also contains a high amount of isoflavones that resemble endogenous estrogens in structure and have estrogenic function by binding to estrogen receptor (ER). We therefore hypothesized that soymilk could have favorable effects on muscle in an estrogen-insufficient condition in females. Here we investigated whether prolonged soymilk intake improves skeletal muscle and satellite cell functions in OVX female mice.

## 2. Materials and Methods 

### 2.1. Animals

The Experimental Animal Care and Use Committee of the Nagasaki University approved animal experimentation. Six-week-old female C57BL/6 mice (Charles River Laboratories, Kanagawa, Japan) were ovariectomized (OVX) under anesthesia. Sham-operated mice were used as a sham-control. One week following the surgery, mice were randomly divided into two groups: one was given the conventional food (control), while the other was given the conventional food replaced by dried soymilk composed of 5% total protein (11.05% of total calorie was replaced by soymilk, termed herein low-soymilk (Low-SM)) (Table S1), for 24 weeks. To further examine the effect of a low amount of soymilk-containing diet on OVX mice, mice were divided into two groups: one was given the conventional food (control) and the other was given the conventional food replaced by dried soymilk composed of 10% total protein (22.7% of total calorie was replaced by soymilk, termed herein high-soymilk (High-SM)) (Table S2), for 12 weeks. The nutritional compositions of dried soymilk were shown in Tables S3 and S4.

### 2.2. Grip Test

Maximal limb muscle force was measured by a Grip Strength Meter (Columbus Instruments, Columbus, OH, USA). Three sets of ten successive measurements were performed to assess limb grip strength. The maximum values in three sets of experiments were used for data analysis.

### 2.3. Isolation and Culture of Myofibers

Satellite cells in their niche on myofibers were isolated from the extensor digitorum longus (EDL) muscle digested by type I collagenase (Worthington Biochemical Corp., Lakewood, NJ, USA) as described previously [20]. Isolated myofibres were then cultured in mitogen-rich medium Dulbecco’s Modified Eagle Medium (DMEM) supplemented with 10% horse serum, 0.5% chicken embryo extract, and 1% penicillin-streptomycin) at 37 °C with 5% CO_2_ for 3 days.

### 2.4. Immunostaining

For immunocytochemistry, satellite cells associated with myofibers were fixed in 2% paraformaldehyde for 10 min at room temperature. Samples were then incubated with primary antibodies at 4 °C overnight following blocking/permeabilization with phosphate-buffered saline containing 0.3% Triton X-100 (Wako Pure Chemical Industries Ltd., Osaka, Japan) and 5% goat serum for 20 min at room temperature. For immunohistochemistry, muscle tissues were isolated from the tibialis anterior (TA) muscle, immediately frozen in 2-methylbutane cooled in liquid nitrogen, and stored at −80 °C. Frozen cross-sections of the TA muscle were fixed in 4% paraformaldehyde and blocked with a DAKO blocking reagent (DAKO Japan, Kyoto, Japan), and incubated with primary antibodies at 4 °C overnight. Immunostained samples were visualized using appropriate species-specific Alexa Fluor 488 or 568 fluorescence-conjugated secondary antibodies (Thermo Fisher Scientific, Tokyo, Japan). Samples were viewed on an all-in-one fluorescence microscope (Keyence, Osaka, Japan) or an Olympus IX83 microscope (Olympus, Tokyo, Japan). Images were optimized globally and assembled into figures using Adobe Photoshop CS5.1 (Adobe Systems Inc., San Jose, CA, USA).

### 2.5. Statistical Analysis

Significant differences were determined using the Student *t*-test. A *p*-value of less than 0.05 was considered as statistically significant. All data are the means ± SEM.

## 3. Results

### 3.1. Muscle Strength and Fiber Sizes Were Unchanged by Prolonged Intake of a Low Amount of Soymilk in OVX Female Mice

**Figure 1:**
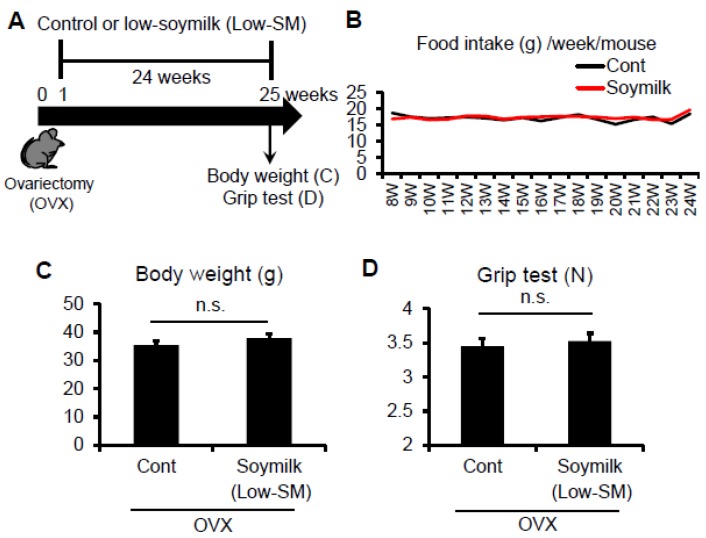
Effect of Low-SM intake on grip strength in OVX mice. (**A**) Six-week-old female C57BL/6 mice were ovariectomized (OVX) and fed with low-soymilk (Low-SM) for 24 weeks, started from 1 week after ovariectomy. Muscle strength was measured by a grip strength meter. Food intake (**B**), body weight (**C**), muscle strength (**D**) were shown (control, *n* = 9; soymilk, *n* = 10 mice). Data represent the mean ± SEM. n.s., not significant.

**Figure 2:**
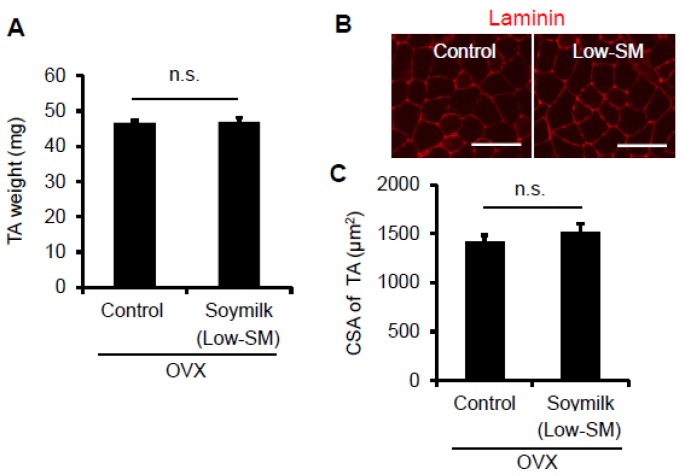
Effect of Low-SM intake on muscle in OVX mice. OVX mice were fed with Low-SM for 24 weeks as shown in Figure 1. (**A**) Muscle weight of TA was measured at 24 weeks followed by the start of soymilk intake. (**B**) Representative images of immunohistochemistry for laminin. (**C**) Cross-sectional area (CSA) of TA muscle (at least 1200 myofibers per mouse were counted). (Control, *n* = 5; soymilk, *n* = 5 mice). Data represent the mean ± SEM. n.s., not significant. An asterisk denotes a significant difference from control (* *p* < 0.05). Scale bar, 100 μm.

In the previous study, we showed that estrogen insufficiency resulted in a decrease in muscle force generation and induced muscle atrophy in a time-dependent manner [8]. Here we examined the effect of soymilk on skeletal muscle in a low estrogen state. C57BL/6 female mice were ovariectmised (OVX) at 6 weeks of age, and then divided into two groups: one received conventional food (control) and the other received dried soymilk composed of 5% total protein (Low-SM) (Supplementary Materials Table S1) for 24 weeks (Figure 1A). We found no significant differences in food intake (Figure 1B), body weight (Figure 1C) and grip strength (Figure 1D) between the control and Low-SM groups. We further confirmed that both muscle wet weight (Figure 2A) and cross-sectional area (CSA) (Figure 2B,C) of TA muscle were unchanged in the Low-SM group compared with those of the control.

### 3.2. Muscle Weakness Was Improved by Prolonged Intake of a High Amount of Soymilk in OVX Mice

**Figure 3:**
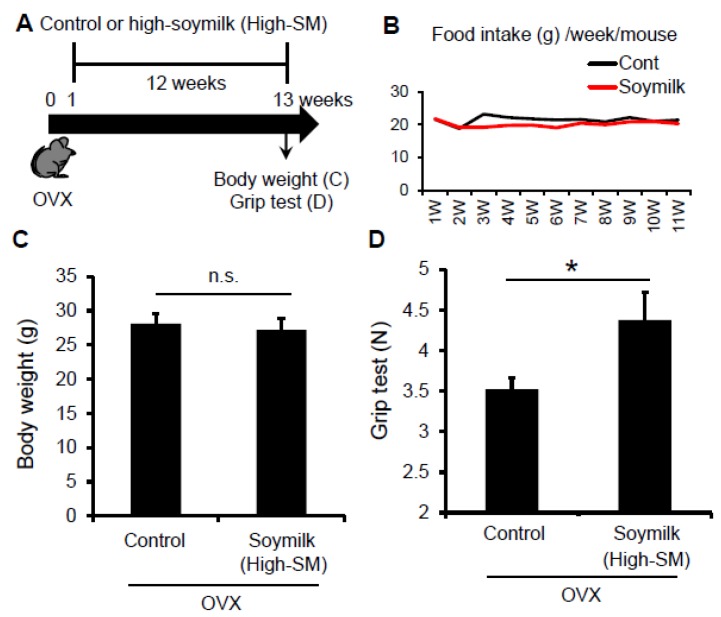
Effect of High-SM intake on grip strength in OVX mice. (**A**) OVX mice were fed with high soymilk (High-SM) for 12 weeks. Muscle strength was measured by a grip strength meter at 12 weeks followed by the start of soymilk intake. Food intake (**B**), body weight (**C**), muscle strength (**D**) were shown (control, *n* = 7; soymilk, *n* = 8 mice). Data represent the mean ± SEM. n.s., not significant. An asterisk denotes a significant difference from control (* *p* < 0.05).

Given that Low-SM did not influence muscle strength and fiber-sizes in OVX mice, we next tested whether a higher amount of soymilk-containing diet could prevent the estrogen insufficiency-induced muscle weakness. According to the experimental model shown in Figure 1, C57BL/6 female mice were ovariectmised at 6 weeks of age, and then divided into two groups: one received control conventional food (control) and the other received dried soymilk composed 10% of total protein (High-SM) (Supplementary Materials Table S2) for 12 weeks (Figure 3A). Food intake (Figure 3B) and body weight (Figure 3C) were unchanged between groups. We found that grip strength was significantly increased in the High-SM group compared with the control (Figure 3D).

**Figure 4:**
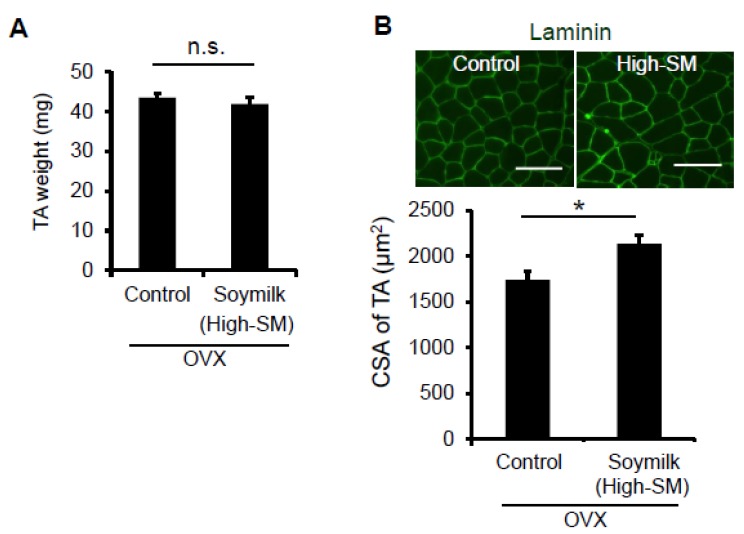
Effect of High-SM intake on muscle in OVX mice. OVX mice were fed with High-SM as shown in Figure 3. (**A**) Muscle weight of TA was measured at 12 weeks followed by the start of soymilk intake. (**B**) Representative images of immunohistochemistry for laminin. CSA of TA muscle was shown (control, *n* = 6; soymilk, *n* = 6 mice). Data represent the mean ± SEM. n.s., not significant. An asterisk denotes a significant difference from control (* *p* < 0.05). Scale bar, 100 μm.

We next determined whether myofiber-sizes were recovered in OVX mice fed with High-SM. Despite no change in muscle wet weight (Figure 4A), the CSA of TA muscle was significantly increased in the High-SM group compared with the control (Figure 4B). However, we failed to demonstrate the favorable effect of High-SM on grip strength in sham-operated female mice (Figure S1), indicating that the beneficial effect of soymilk on muscle function is limited to the female mice with a low estrogen state. Thus, our results suggested that prolonged intake of a high amount of soymilk alleviates muscle weakness in estrogen-insufficient mice.

### 3.3. Soymilk Reserves Muscle Stem Cell Function in OVX Mice

Muscle satellite cells play a crucial role in providing myonuclei for muscle repair and regeneration in adult muscle [21,22]. Our previous study revealed that prolonged estrogen-insufficient state affected satellite cell function and muscle regeneration in OVX female mice [8]. In the current study, we tested if High-SM could improve satellite cell function as well as muscle strength and myofiber-sizes in OVX mice.

**Figure 5:**
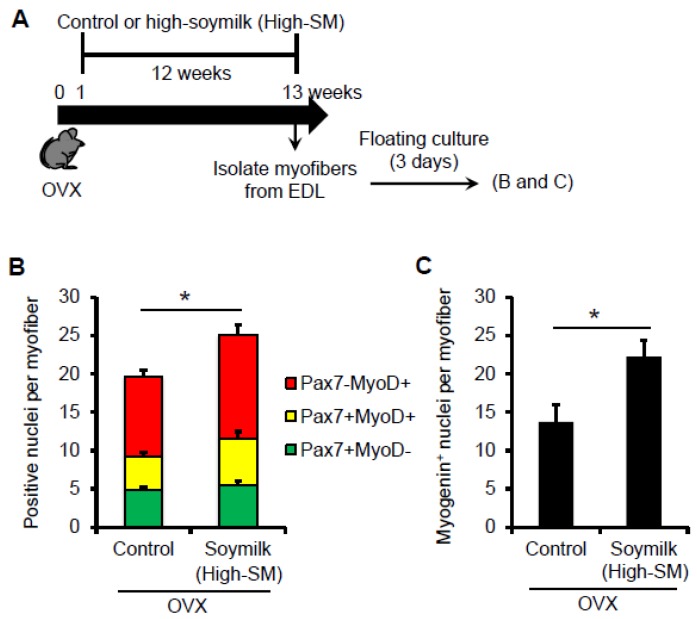
Effect of High-SM intake on satellite cell function in OVX mice. (**A**) OVX mice were fed with High-SM as shown in Figure 3. Satellite cells retained in their niche on myofibers were isolated from the extensor digitorum longus (EDL) muscle in OVX mice, cultured in mitogen-rich medium for 72 h, and co-immunostained for Pax7 and MyoD. Number of Pax7^+^ and/or MyoD^+^ (**B**) or Myogenin^+^ (**C**) nuclei per myofibre was quantified. (more than 20 myofibers per mouse were counted; control, *n* = 5; soymilk, *n* = 4 mice). Data represent the mean ± SEM. n.s., not significant. An asterisk denotes a significant difference from control (* *p* < 0.05).

Satellite cells are normally quiescent but are activated following muscle injury. Activated satellite cells proliferate and differentiate to provide new myonuclei. Satellite cells retained in their niche on myofibers were isolated from the extensor degitorum longus (EDL) muscles. Satellite cells associated with myofibers were then cultured in mitogen-rich medium for 3 days (Figure 5A). It is important to note that, in our culture condition, the mitogen-rich medium contains estrogens [23] and thus similarly influences the satellite cells isolated from OVX mice. Co-immunostaining for the transcription factors Pax7 and MyoD, which mark nearly all the satellite cell-populations in the states of activation/proliferation (Pax7+MyoD+), differentiation (Pax7-MyoD+) and self-renewal (Pax7+MyoD-)[24,25]. There was no alteration in the number of Pax7^+^ quiescent satellite cells per freshly isolated myofiber between the control and High-SM groups (data not shown), whereas cultured myofibers showed that the total number of satellite cells (Pax7^+^ and/or MyoD^+^) (Figure 5B) and Myogenin+ differentiating cells (Figure 5C) per myofiber were significantly increased in High-SM group. Indeed, these data indicated that prolonged intake of a high amount of soymilk recovered the abilities in proliferation and differentiation in satellite cells in OVX mice.

## 4. Discussion

Although there is increasing interest in exploring the effects of HRT on muscle mass and strength, HRT for postmenopausal women may be associated with serious risks of coronary heart disease and breast cancer [12,13]. Soy isoflavones are a promising alternative for HRT in postmenopausal women. Our previous study revealed that estrogen insufficiency resulted in muscle atrophy and reduced muscle force generation in young OVX female mice [8]. Here we demonstrated that the decrease in myofiber-sizes and force generation in OVX mice could be improved with prolonged soymilk intake. Our results also showed that a low amount of soymilk-containing diet was insufficient to ameliorate muscle weakness in OVX mice, suggesting that a certain amount of soymilk was needed to preserve muscle function in our mouse model.

Accumulating evidence indicates the importance of estrogens on muscle repair and regeneration after injury. Treatment with 17beta-estradiol promotes activation and proliferation of satellite cells and stimulates muscle growth and regeneration [11,26,27,28]. Our previous study also demonstrated that population expansion was markedly impaired in satellite cells in their niche on myofibers isolated from OVX mice ex vivo [8]. Indeed, the intrinsic function of satellite cells is declined by estrogen insufficiency in female mice. In the current study, we showed that soymilk diet mitigated dysfunction of satellite cells in a low estrogen state. These findings suggest that dysfunctions in both muscle and its resident stem cells in OVX mice could be improved by prolonged soymilk intake.

Isoflavone aglycones including daidzein and genistein can be metabolized by the gut microflora to produce equol, an estrogenic metabolite from daidzein [29,30,31]. Equol is structurally similar to the 17beta-estradiol, and mimics the action of estrogens through binding to estrogen receptors. Although there is a significant interindividual difference in humans, most animals including mice retain the ability to produce equol when fed soy products [32]. In this study, we did not assess the levels of equol in the blood of mice and what ingredients in soymilk ameliorated OVX-induced muscle weakness. Studies reported that soymilk contains the soybean trypsin inhibitor and the Bowman–Birk protease inhibitor [33,34]. These protease inhibitors suppress the protease-activated receptor 2 cleavage, influence cytoskeletal and cell surface organization, and reduce keratinocyte phagocytosis, resulting in an induction of skin depigmentation [35]. The Bowman–Birk protease inhibitor has an anti-inflammatory effect [36] and is also known to act as an anti-carcinogenic factor [37]. Considering these aspects, isoflavone-independent mechanisms might be involved in the preferable effect of soymilk on muscle function in OVX mice.

Our ovariectomy model displayed a significantly atrophic uterus and increased body weight, which partially mimicked a postmenopausal state [8]. In the current study, although uterine wet weight was slightly increased by soymilk intake in OVX mice, its improvement efficiency was far less compared with 17beta-estradiol-treated OVX mice (data not shown). Indeed, it is unlikely that soymilk alone is an alternative agent for recovery of reproductive function in a state of estrogen-insufficiency. Our findings also showed that soymilk diet did not upregulate grip strength in the sham-operated female mice. Thus, prolonged soymilk intake can prevent muscle weakness in a low estrogen state but it does not seem to gain muscle strength in healthy young female mice. According to our calculations, the amount of High-SM in our mouse model (30 g body weight) would be equivalent to approximately 800 mL of soymilk (10% soybean solid content) intake a day for humans (60 kg body weight).

## 5. Conclusions

In conclusion, we demonstrated for the first time that soymilk exerts a favorable effect on muscle weakness in OVX mice. Therefore, soymilk may be applicable to effective and inexpensive natural foods and alternative for HRT, for musculoskeletal care and management in female athletes with menstrual dysfunction as well as post-menopausal woman. However, it remains unclear how prolonged soymilk intake ameliorated muscle and satellite cell functions. Further studies are needed to identify soymilk-derived factor(s) responsible for improving the OVX-induced muscle weakness.

## Supplementary Materials

The following are available online at www.mdpi.com/2072-6643/9/8/834/s1, Table S1: Ingredient of Low-SM, Table S2: Ingredient of High-SM, Table S3: Composition of powdered soymilk (Low-SM), Table S4: Composition of powdered soymilk (High-SM), Figure S1: Effect of High-SM on muscle strength in sham-operated mice.
