# Supplementary material for: Soymilk Improves Muscle Weakness in Young Ovariectomized Female Mice
Source: Nutrients. 2017 Aug 4;9(8):834. doi: 10.3390/nu9080834 (PMC5579627; doi:10.3390/nu9080834)
Supplement: Supplementary file 1 [file nutrients-09-00834-s001.docx]

**Table S1.** Ingredient of Low-SM.

| **Ingredient** | **Control** | **Low-SM** |
| --- | --- | --- |
| Casein (%) | 20.00 | 14.19 |
| Cornstarch (%) | 39.75 | 38.87 |
| Dextrized cornstarch (%) | 13.20 | 12.91 |
| Sucrose (%) | 10.00 | 10.00 |
| Soybean oil (%) | 7.00 | 3.20 |
| Cellulose (%) | 5.00 | 4.74 |
| Mineral mix (%) | 3.50 | 3.50 |
| Vitamine mix (%) | 1.00 | 1.00 |
| L-Cystine (%) | 0.30 | 0.30 |
| Choline bitartate (%) | 0.25 | 0.25 |
| tert-Butylhydraquinone (%) | 0.0014 | 0.0014 |
| Powdered soymilk (%) | 0.00 | 11.05 |
| Total (%) | 100.00 | 100.00 |
| Energy (kcal/100 g) | 372.20 | 370.54 |

**Table S2.** Ingredient of High-SM.

| **Ingredient** | **Control** | **High-SM** |
| --- | --- | --- |
| Casein (%) | 20.00 | 8.40 |
| Cornstarch (%) | 39.75 | 37.08 |
| Dextrized cornstarch (%) | 13.20 | 12.31 |
| Sucrose (%) | 10.00 | 10.00 |
| Soybean oil (%) | 7.00 | 0.00 |
| Cellulose (%) | 5.00 | 4.46 |
| Mineral mix (%) | 3.50 | 3.50 |
| Vitamine mix (%) | 1.00 | 1.00 |
| L-Cystine (%) | 0.30 | 0.30 |
| Choline bitartate (%) | 0.25 | 0.25 |
| tert-Butylhydraquinone (%) | 0.0014 | 0.0014 |
| Powdered soymilk (%) | 0.00 | 22.70 |
| Total (%) | 100.00 | 100.00 |
| Energy (kcal/100 g) | 372.20 | 375.57 |

**Table S3.** Composition of powdered soymilk (Low-SM)

| Component | % | Calorie (kcal) |
| --- | --- | --- |
| Water | 3 | - |
| Protein | 45 | 181 |
| Fat | 36 | 327 |
| Carbohydrate | 8 | 32 |
| Dietary Fiber | 2 | 2 |
| Energy | - | 543 |

**Table S4.** Composition of powdered soymilk (High-SM)

| Component | % | Calorie (kcal) |
| --- | --- | --- |
| Water | 3 | - |
| Protein | 44 | 176 |
| Fat | 38 | 339 |
| Carbohydrate | 8 | 32 |
| Dietary Fiber | 2 | 2 |
| Energy | - | 550 |


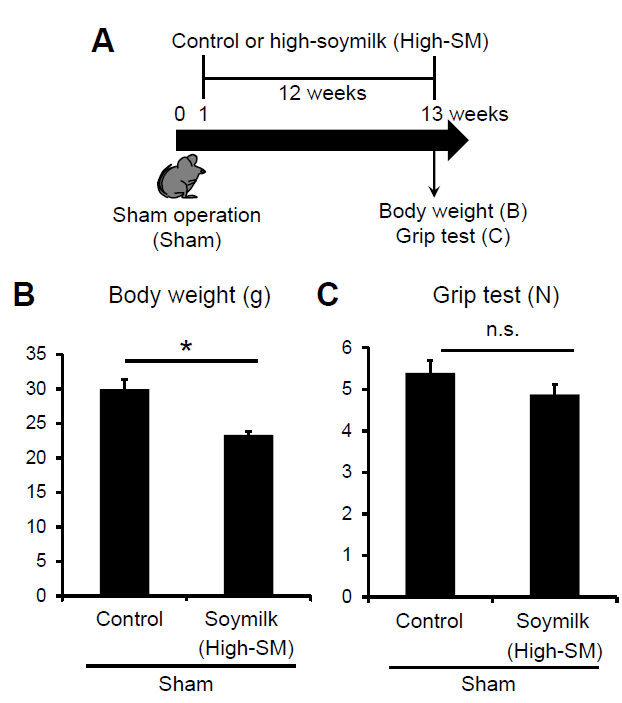


**Figure S1. Effect of High-SM on muscle strength in sham-operated mice.** (**A**) Six-week-old female C57BL/6 mice were sham-operated and fed with High-SM for 12 weeks, started from 1 week after sham-operation. Body weight (**B**) and muscle strength (**C**) measured by a grip strength meter were shown (control, *n* = 6; soymilk, *n* = 6 mice). Data represent the mean ± S.E.M. n.s., not significant.
